# Supplementary material for: The transcriptional and translational outcomes for pseudogenes in bacterial endosymbionts
Source: Mol Biol Evol. 2026 Jun 23;43(7):msag153. doi: 10.1093/molbev/msag153 (PMC13345370; doi:10.1093/molbev/msag153)
Supplement: msag153_Supplementary_Data [file msag153_supplementary_data.zip › 02_21_RibosomeRescue-June2026-SupplementalFigures.docx]

supplemental figures


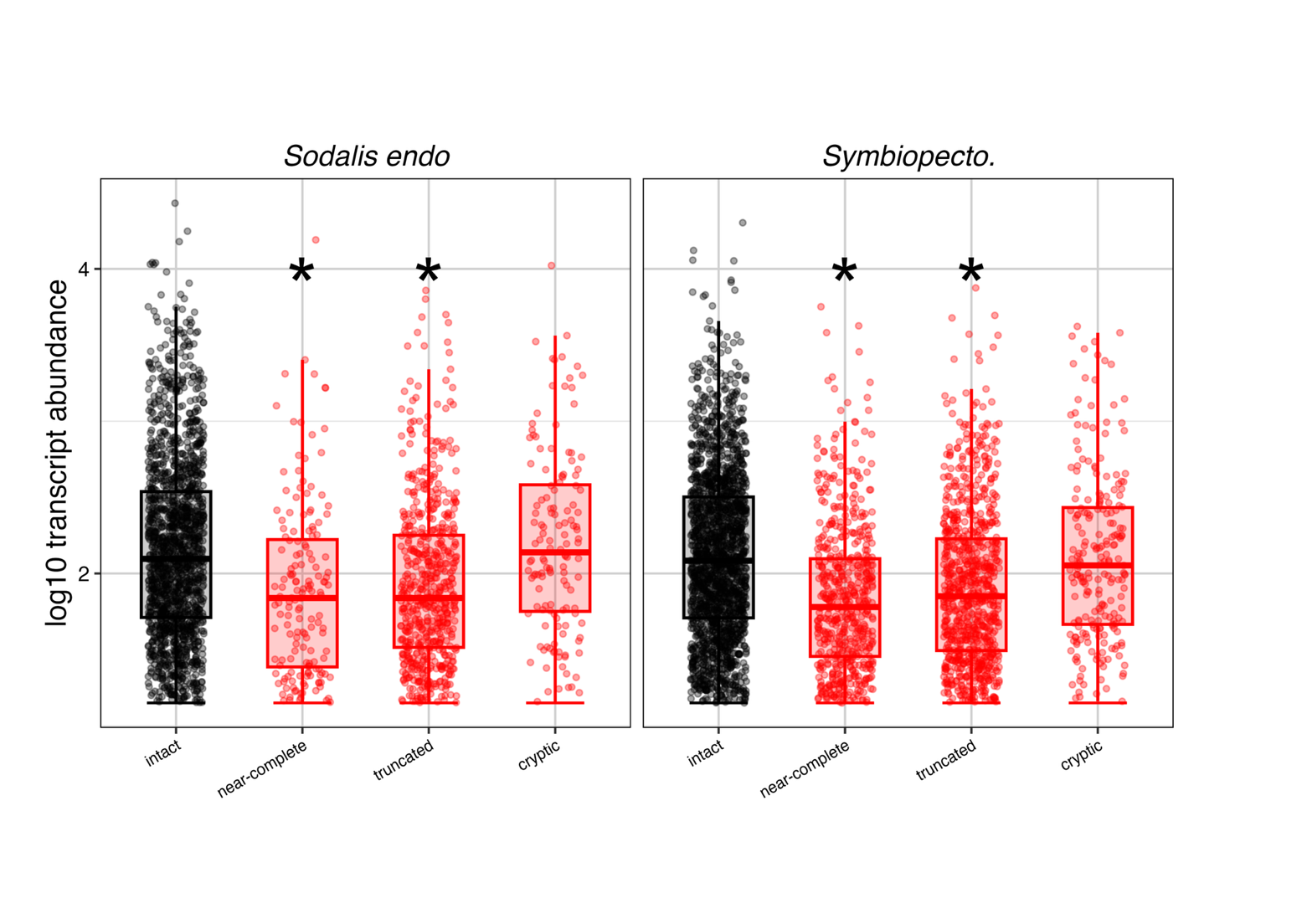


**Supplemental Figure 1**: Transcript levels in *Sodalis endo* and *Symbiopectobacterium endo* of intact genes (black dots) and the different categories of pseudogenes (red dots). Welch’s t-test was applied to compare the expression profiles of the different types of pseudogenes with those of intact genes. Asterisks indicate where significant differences were predicted (p-value < 1.35E-06).


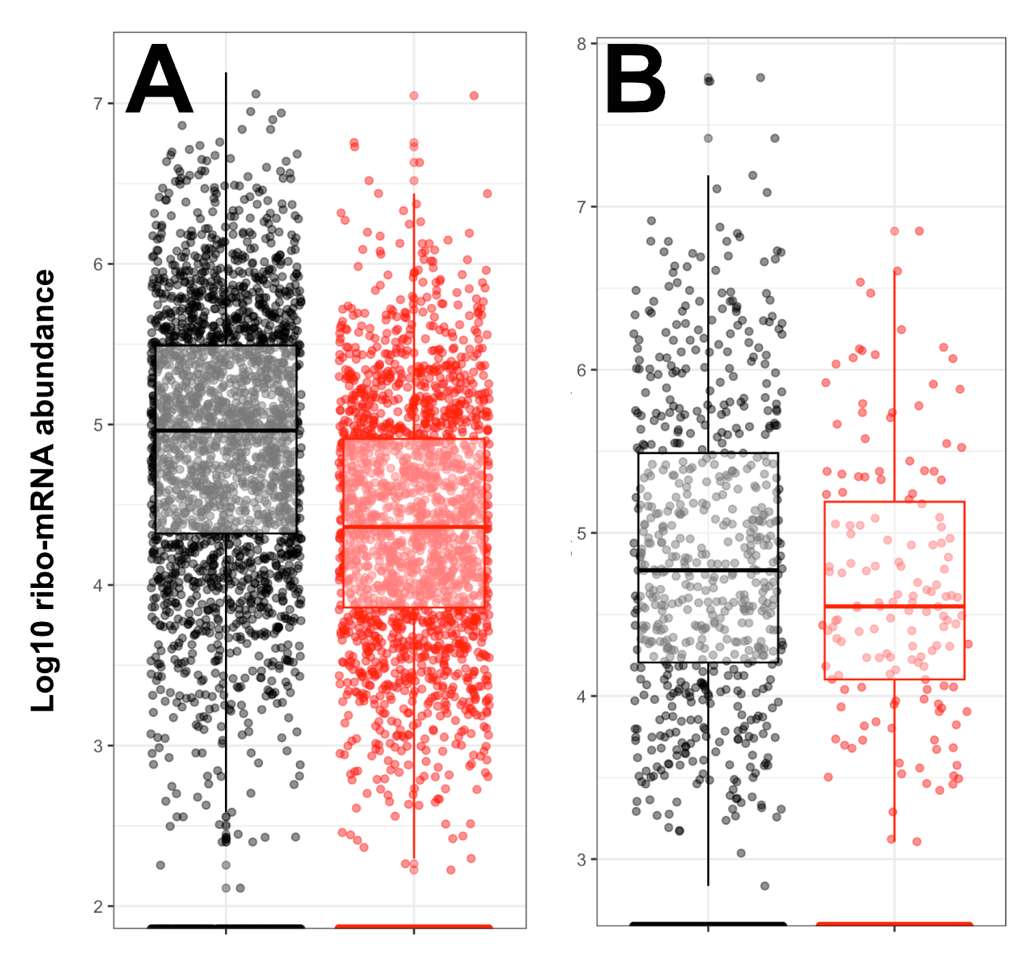


**Supplemental Figure 2**: Normalized RNA levels among the ribosomes in A) *Symbiopectobacterium endo*. and B) *Sodalis endo*. Pseudogenes are colored red.


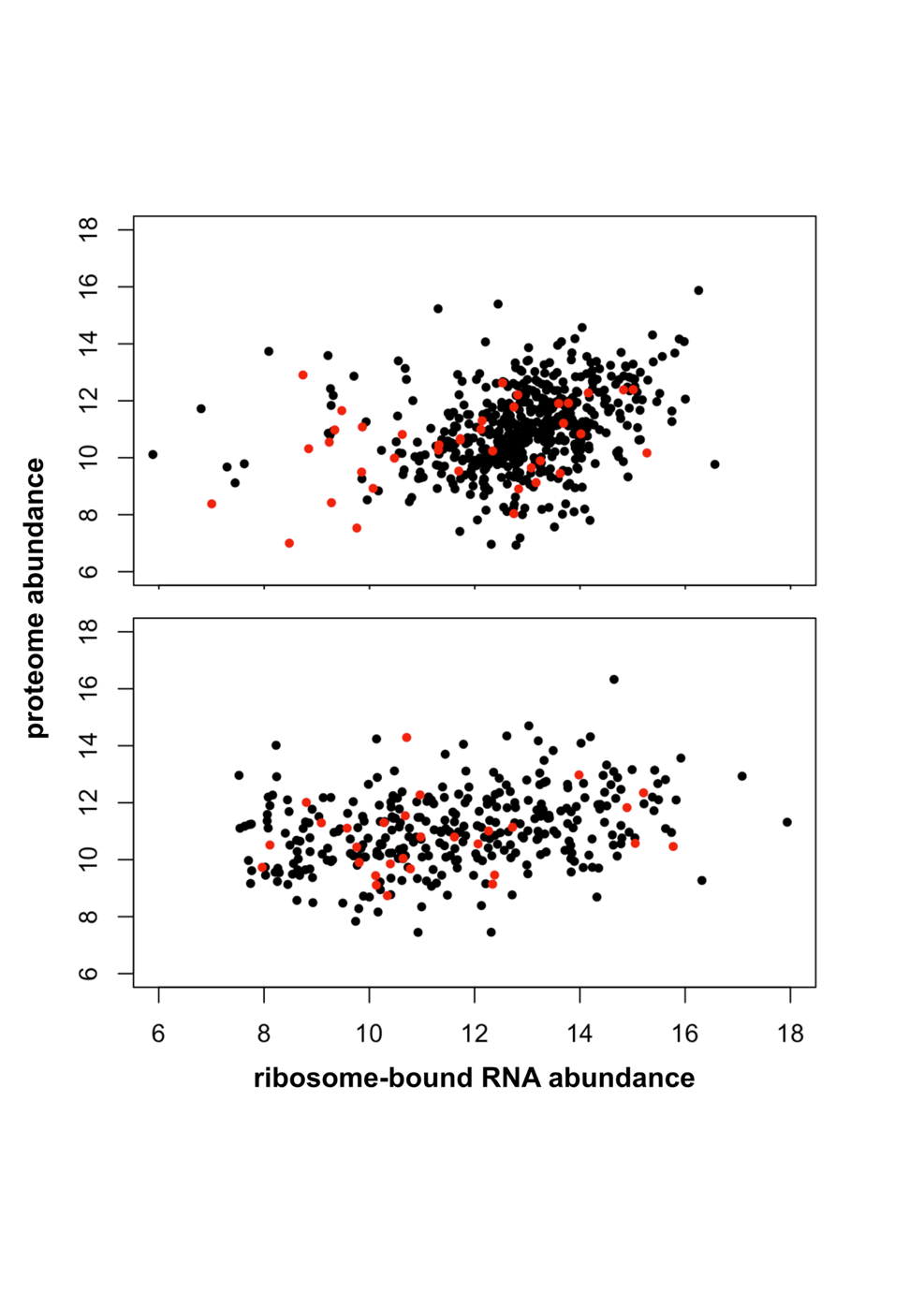


**Supplemental Figure 3**: Comparison of ribosome-bound RNA levels with protein abundance in *Symbiopectobacterium endo*. (top) and *Sodalis endo*. (bottom). Abundances are shown on a normalized log2 scale, with pseudogenes colored red.


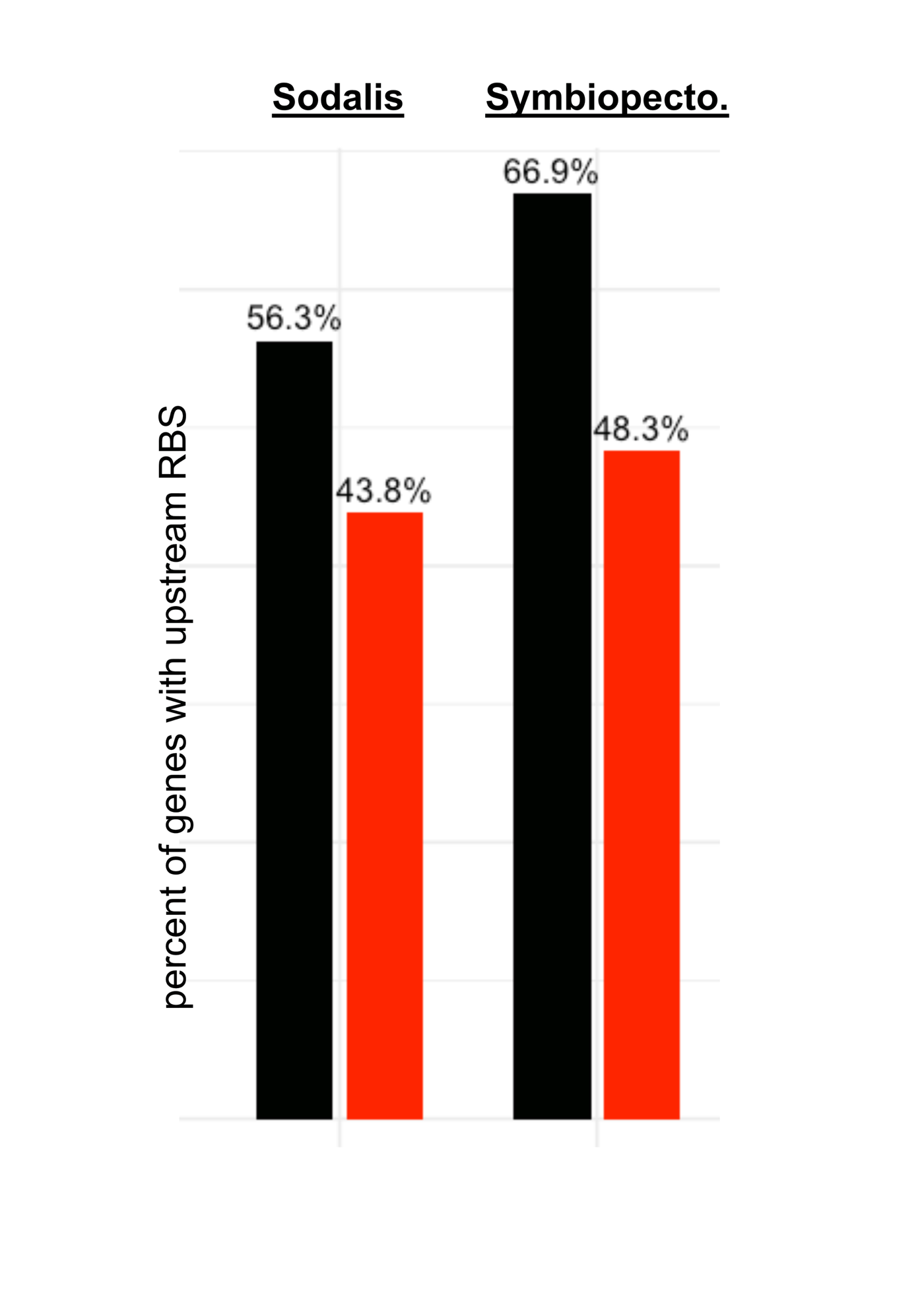


**Supplemental Figure 4**: Percentage of intact (black) and pseudogenes (red) with upstream ribosomal binding sites, as predicted via Prodigal.


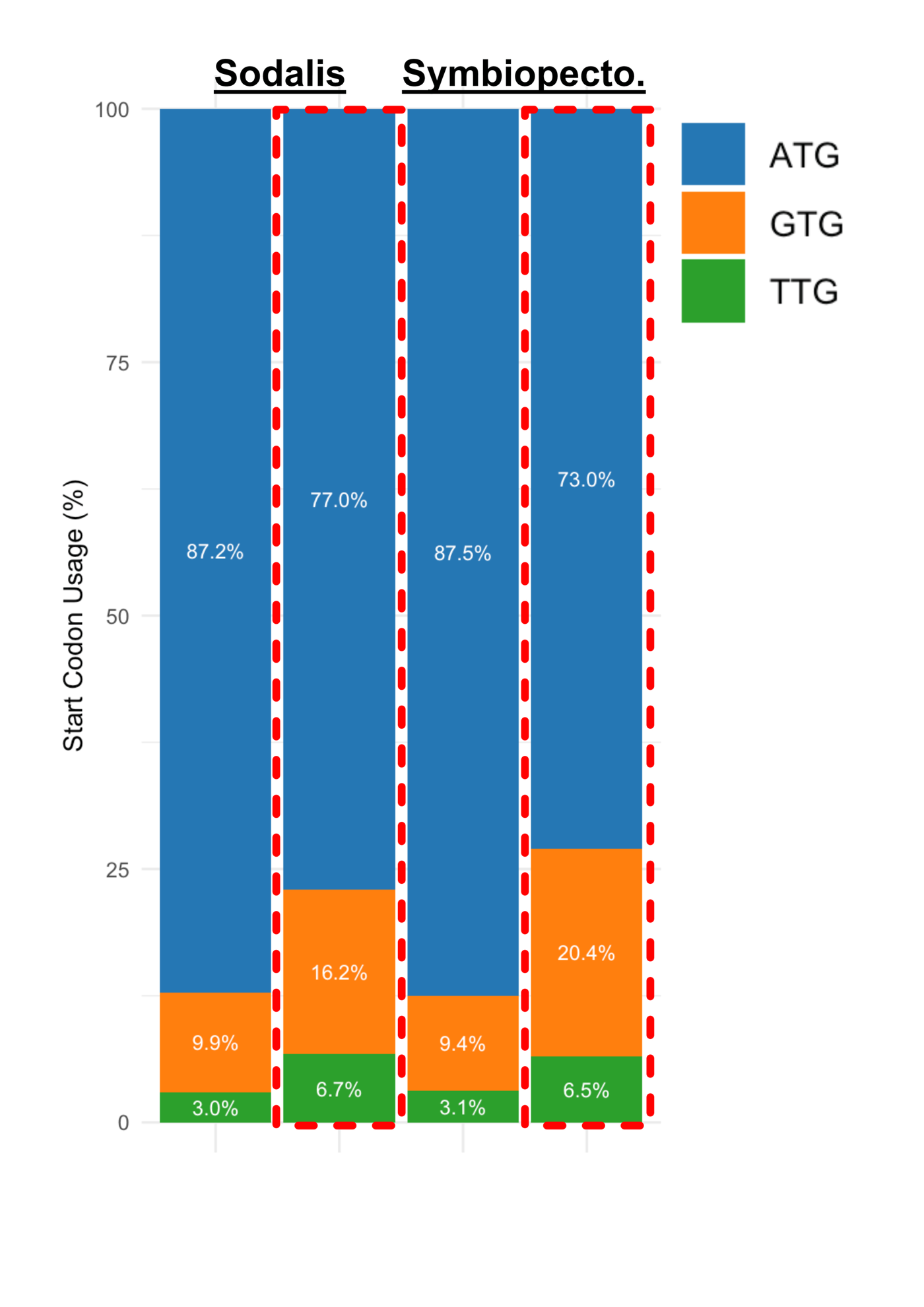


**Supplemental Figure 5**: Percent of intact genes and pseudogenes (enclosed in dotted red boxes) that start with each of three types of start codons.


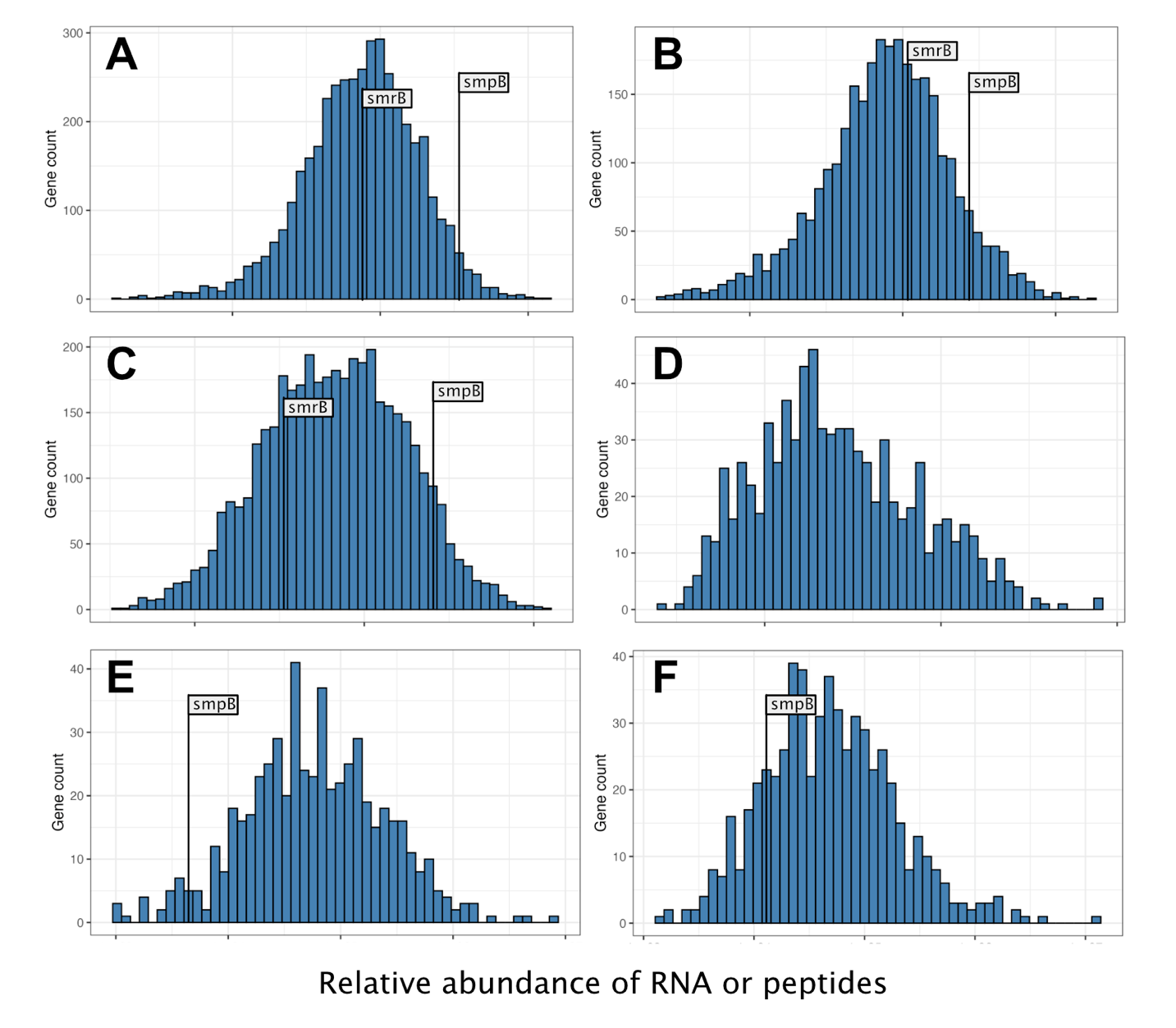


**Supplemental Figure 6:** *Symbiopectobacterium endo*. and *Sodalis endo*, with an emphasis on where in each distributions the protein or transcript levels for *smpB* and *smrB* fall. Top row shows relative levels among whole transcriptomes in A) *Symbiopectobacterium* and B) *Sodalis endo*. Middle row shows relative levels among ribosome-copurified RNA in C) *Symbiopectobacterium* and D) *Sodalis endo*. Bottom row shows relative protein abundance levels in E) *Symbiopectobacterium* and F) *Sodalis endo*. Neither *smpB* nor *smrB* transcripts were detected among the *Sodalis endo*. ribosome-copurified RNA (panel D).


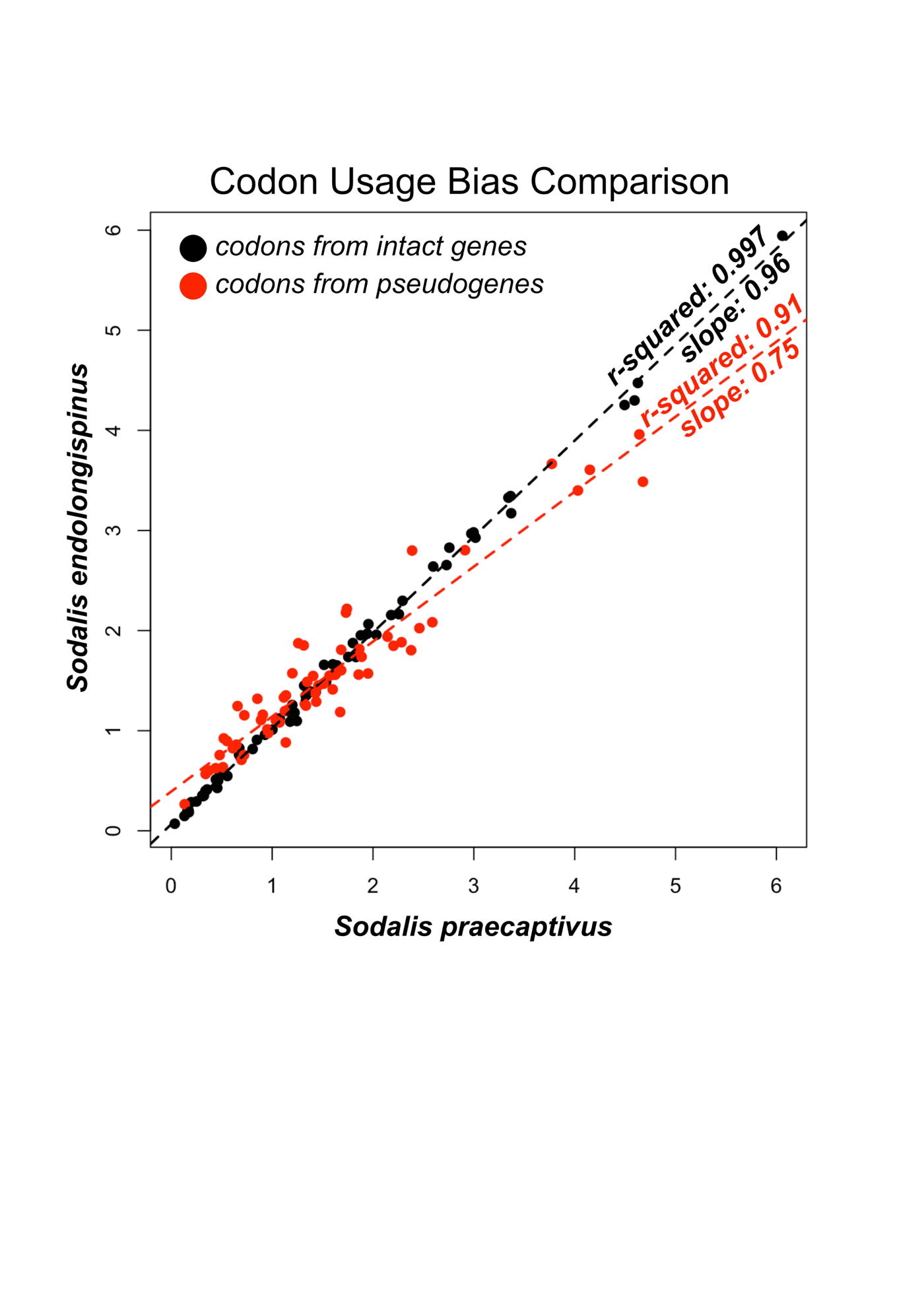


**Supplemental Figure 7**: Comparison of codon usages between *Sodalis endo*. and its closest free-living relative *Sodalis praecaptivus*. Each black dot corresponds to a codon, with its frequency among the intact genes in *S. praecaptivus* shown along the x-axis and its frequency among Sodalis endo. shown along the y-axis. Codons from pseudogenes in *Sodalis endo*. are shown in red on the same scale. Linear model results superimposed over each regression.


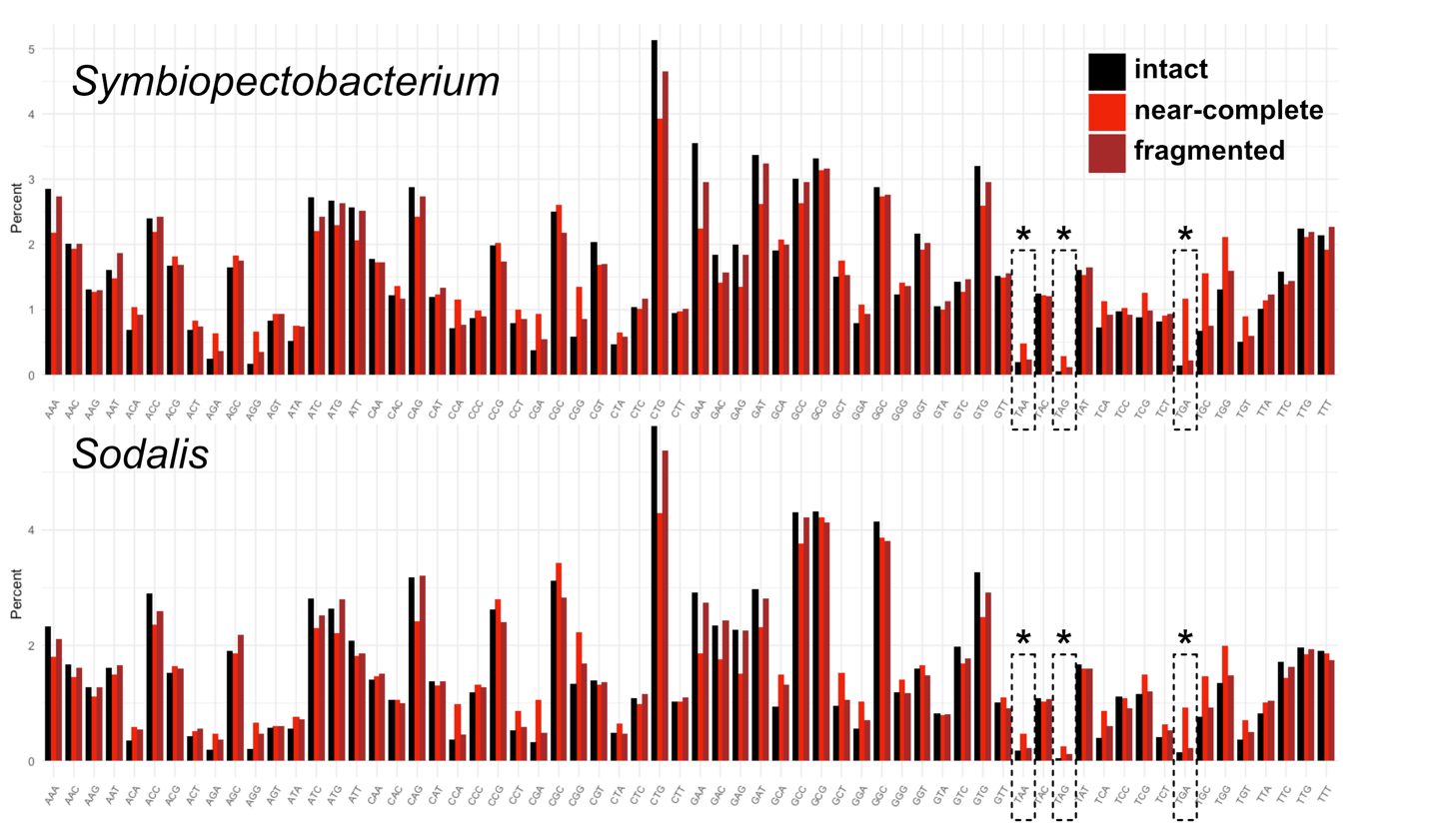


**Supplemental Figure 8** : Codon usage percentages across all intact genes (black), in comparison with two types of pseudogenes: near-complete and truncated. Y-axis shows percent across all codons (within-category). Highlighted with asterisks are stop codons TAG, TGA, and TAA.


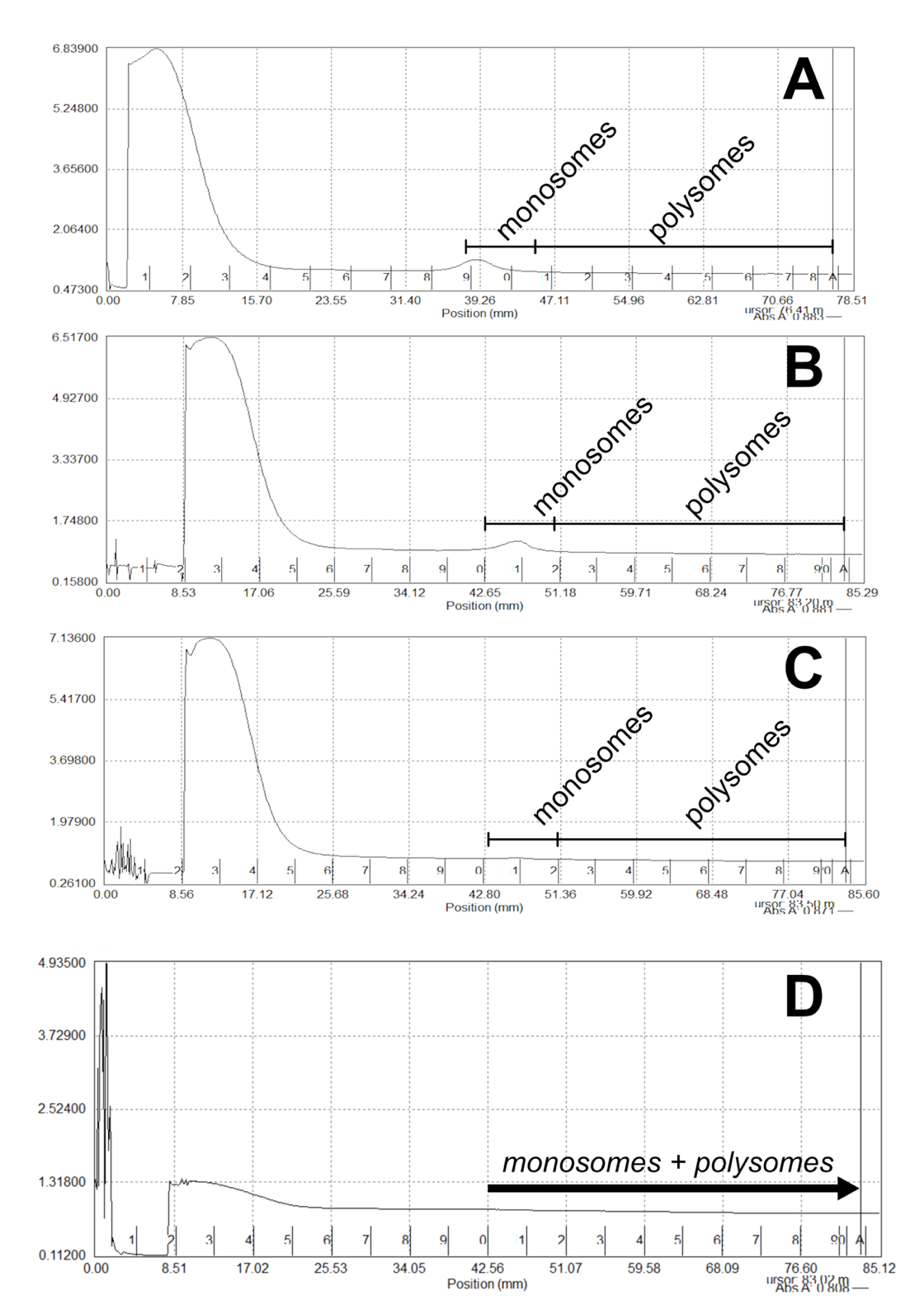


**Supplemental Figure 9**: Absorbance measurements (A260), shown on the y-axis, taken during fractionation of whole-insect *Pseudococcus longispinus* samples (A-C) and P. longispinus bacteriomes (D). Fraction numbers are shown on the x-axis, with the top of the centrifuge tube corresponding to fraction 1 and the bottom (including pellet) corresponding to fraction 20. Monosome peaks are visible in panels A and B starting at fraction 10, which is the highest fraction we used for downstream sequencing and analysis (i.e., we used fractions 10-20 to infer ribosome-bound RNAs).


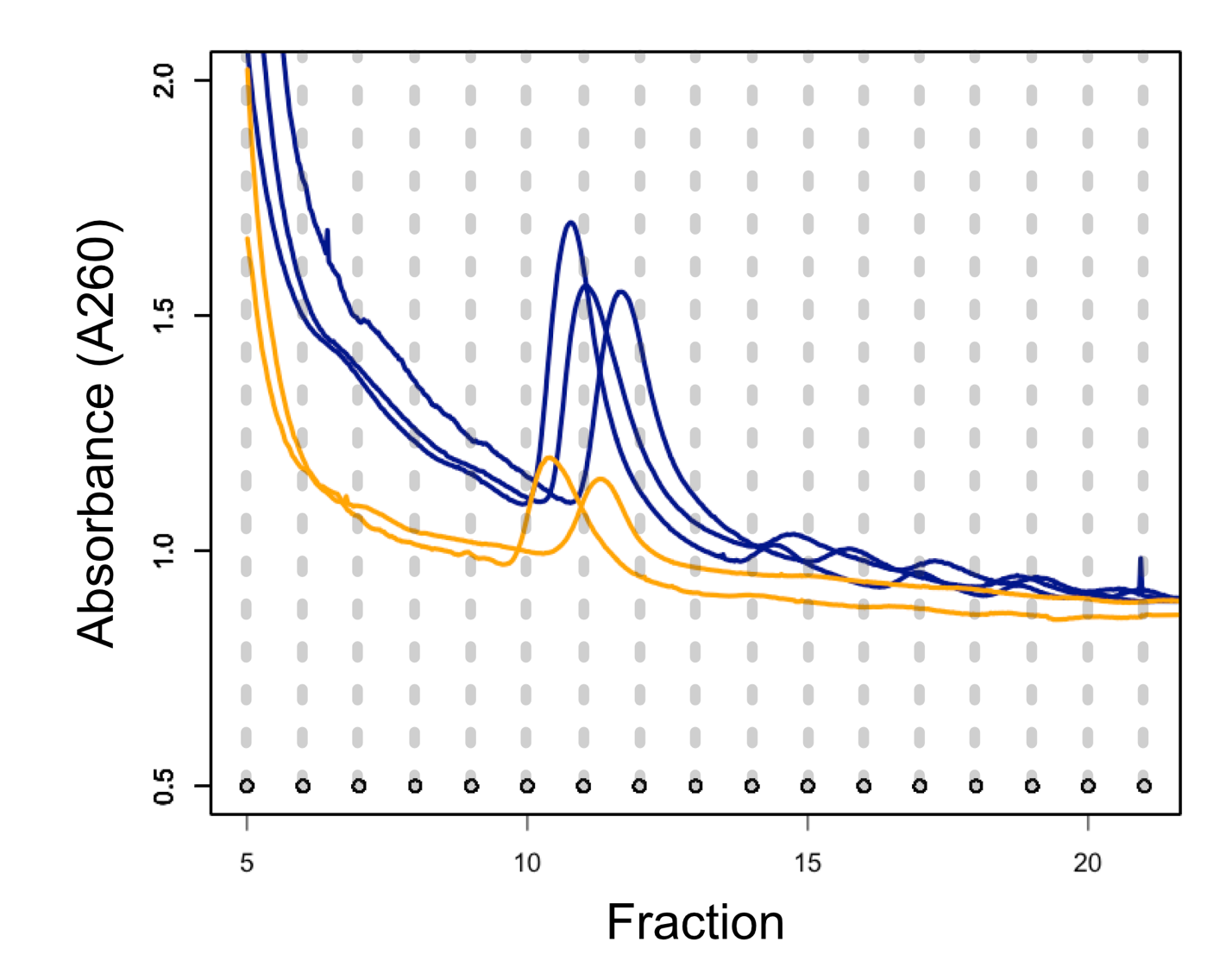


**Supplemental Figure 10**: Absorbance measurements taken during fractionation of a 10-50% sucrose gradient after ultracentrifugation. Only fractions 5-20 are shown. *Sodalis praecaptivus* HS1 samples are shown in blue (triplicate), and two whole-insect *P. longispinus* samples (from panels A and B in Supplemental Figure 9) are shown in orange. Each dot on the bottom corresponds to an individual fraction from the sucrose gradient, collected automatically via the gradient fractionator.
